# Ex Vivo-Generated Tolerogenic Dendritic Cells: Hope for a Definitive Therapy of Autoimmune Diseases

**DOI:** 10.3390/cimb46050249

**Published:** 2024-04-28

**Authors:** Jonny, Enda Cindylosa Sitepu, Chairul A. Nidom, Soetojo Wirjopranoto, I. Ketut Sudiana, Arif N. M. Ansori, Terawan Agus Putranto

**Affiliations:** 1 Indonesia Army Cellcure Center, Gatot Soebroto Central Army Hospital, Jakarta 10410, Indonesia; endacsitepu@gmail.com (E.C.S.);; 2 Faculty of Medicine, University Prima Indonesia, Medan 20118, Indonesia; 3 Faculty of Military Medicine, Indonesia Defense University, Jakarta 16810, Indonesia; 4 Professor Nidom Foundation, Surabaya 60236, Indonesia; nidomca@fkh.unair.ac.id (C.A.N.);; 5 Faculty of Veterinary Medicine, Universitas Airlangga, Surabaya 60115, Indonesia; 6 Faculty of Medicine, Universitas Airlangga, Surabaya 60115, Indonesia; s.tojowirjopranoto@yahoo.com (S.W.); iksudiana57@gmail.com (I.K.S.)

**Keywords:** dendritic cells, immune tolerance, cellular immunotherapy, autoimmune disease, systemic lupus erythematosus

## Abstract

Current therapies for autoimmune diseases are immunosuppressant agents, which have many debilitating side effects. However, dendritic cells (DCs) can induce antigen-specific tolerance. Tolerance restoration mediated by ex vivo-generated DCs can be a therapeutic approach. Therefore, in this review, we summarize the conceptual framework for developing ex vivo-generated DC strategies for autoimmune diseases. First, we will discuss the role of DCs in developing immune tolerance as a foundation for developing dendritic cell-based immunotherapy for autoimmune diseases. Then, we also discuss relevant findings from pre-clinical and clinical studies of ex vivo-generated DCs for therapy of autoimmune diseases. Finally, we discuss problems and challenges in dendritic cell therapy in autoimmune diseases. Throughout the article, we discuss autoimmune diseases, emphasizing SLE.

## 1. Introduction

Autoimmunity is a diverse group of conditions where the body’s immune system mistakenly targets and attacks its own cells, tissues, and organs. From rheumatoid arthritis to lupus, multiple sclerosis to type 1 diabetes, autoimmune diseases encompass a spectrum of disorders, each with its unique set of symptoms, triggers, and complexities. While the precise mechanisms underlying these conditions vary, they often share common features, characterized by inflammation, tissue damage, and impaired function in affected organs [1,2,3,4].

At the core of autoimmune diseases lies a delicate interplay between genetic predisposition, environmental factors, and dysregulated immune responses. Genetic susceptibility may predispose individuals to certain autoimmune conditions, while environmental triggers such as infections, stress, or dietary factors can exacerbate immune dysfunction [5]. The immune system, in its quest to protect, becomes entangled in a state of confusion, launching attacks against self-antigens—normal proteins and tissues—mistakenly identified as foreign invaders.

Management of autoimmune diseases typically involves a multifaceted approach aimed at alleviating symptoms, suppressing immune activity, and preventing further damage to affected tissues. This may entail a combination of medications to modulate immune responses, lifestyle modifications to reduce triggers and promote overall well-being, and, in some cases, therapeutic interventions such as physical therapy or surgery to address specific complications [6].

However, glucocorticoids, which are the most common drugs used to manage autoimmunity, cause many side effects on various organs. The side effects include Hirsutism, moon facies, buffalo hump, acne, striae, and weight gain. The use of glucocorticoids in large doses can also cause myopathy and hyperlipidemia. Long-term use of low doses of glucocorticoids can cause growth inhibition, HPA axis suppression, glucocorticoid-induced osteonecrosis, cataracts, acne, skin lesions, and weight gain. In addition, as a result of nonspecific immunosuppressive abilities, the use of these immunosuppressant agents increases susceptibility to infection [7]. Likewise, cyclophosphamide, which is still a first-line drug widely used to treat patients with Lupus Nephritis, has severe side effects such as myeloid suppression, infection, gonadal toxicity, bladder toxicity, and even cancer [8].

The definitive therapy for autoimmunity is the restoration of immune system tolerance. Therapeutic induction of tolerance makes it possible to ‘reset’ abnormalities in the immune system, theoretically allowing long-term, drug-free remission [9]. Over the last decade, researchers have developed approaches to ex vivo differentiation of ‘tolerogenic’ immune cells, which can then be transferred to the body as a potential route for the induction of therapeutic tolerance. One of the new immunotherapy approaches for autoimmune diseases is autologous dendritic cells (DCs) with tolerogenic function (tolerogenic DC or tolDC) [10,11]. This approach utilizes the capacity of DCs as an antigen-presenting cell to induce antigen-specific tolerance.

Autoreactive cells target specific tissue antigens in type-1 diabetes mellitus (T1DM), rheumatoid arthritis (RA), multiple sclerosis (MS). This is not the case in Systemic lupus erythematosus (SLE) [1,2,3,4]. SLE is an autoimmune disorder characterized by antinuclear antibody (ANA) production due to poor cell apoptosis [12]. ANA targets various tissues throughout the body that cause complex autoimmune diseases with various clinical manifestations affecting multiple organ systems. This has become a unique challenge in developing antigen-specific treatment of SLE. Furthermore, despite promising results in several preclinical studies, no experiments on SLE patients have been reported.

Therefore, this article aims to summarize the conceptual framework for developing DC-based strategies for autoimmune diseases, emphasizing the potential application of SLE therapy. We explain the basic science in tolerance establishment, focusing on the role of DCs and its contribution to the pathogenesis of autoimmunity. From there, we discuss the latest developments, problems, and challenges in dendritic cell therapy in autoimmune diseases, focusing on SLE.

## 2. The Role of Dendritic Cells in Autoimmunity

Abnormalities in immunological tolerance can cause activation of unwanted immune responses to self-antigens, causing autoimmune diseases, such as SLE, type-1 diabetes mellitus (T1DM), rheumatoid arthritis (RA), multiple sclerosis (MS), and inflammatory bowel disease (IBD). Studies in autoimmune patients show that aberrations of immune cell activation, including lymphoid and myeloid cells, cause inflammation in the target organs [13,14]. Currently, the primary treatment of autoimmune diseases is the administration of immunosuppressive agents, but the administration of these drugs often causes side effects such as infection and cancer [15,16,17]. In addition, accumulating evidence has shown that DCs have a central role in maintaining a balance of central or peripheral tolerance [18].

Inducing a primary immune response, the DC acts as a professional antigen-presenting cell that bridges innate and adaptive immunity. DCs are distributed throughout the body, including lymphoid and non-lymphoid organs. DCs can be classified into conventional DCs (cDCs) and non-conventional DCs consisting of plasmacytoid DCs (pDCs) and monocyte DCs (MoDCs) [19]. The pDC is characterized by high expression of CD123, BDCA-2 (CD303), BDCA4 (CD304) and low expression of CD11c [20]. The pDC is in charge of secreting Interferon (IFN) type I in response to viral infection and exposing antigens to CD8+ T cells. In conditions where immunosensing is excessive, IFN-mediated autoimmunity can occur [20,21]. Meanwhile, the cDC and MoDC play a role in forming central and peripheral immune tolerance [22].

The DC in an immature state, or referred to as immature DC (iDC), will continue to patrol the entire body. Most are in peripheral tissues (e.g., liver, kidney, intestines, and skin) and secondary lymphoid organs. iDCs can recognize a large number of pathogen-associated molecular patterns (PAMPs) and danger-associated molecular patterns (DAMPs) through pattern recognition receptor (PRR), Toll-like receptor (TLR), or C-type lectin receptor [23]. iDC highly expresses PRR and major histocompatibility complex class II (MHC-II), while CD80 and CD86 expressions are low. In addition, iDC has low lysosomal activity [24]. iDC processes the encountered antigens into smaller peptides to be presented on the cell surface via MHC class I/II [25]. Exposure to antigens triggers iDC maturation so that the ability to process new peptides is lost, and the ability to present antigens to T cells arises [26]. The mature DC (mDC) expresses MHC and stimulation molecules (CD40, CD80, and CD86), secretes proinflammatory cytokines (IL-1β, IL-6, IL-12), and tumor-necrosis factor α (TNF-α), increases CCR7 and CXCR4 expression, allowing these cells to migrate to lymph nodes. For T cell activation to be effective, three signals are required: (i) interaction between TCR (T Cell Receptor) and antigen/MHC complex; (ii) interaction between CD28 and co-stimulating molecules (CD80 or CD86); and (iii) cytokine and chemokine secretion [27].

In addition to inducing immune responses to pathogens and foreign antigens, the DC is an essential modulator of central and peripheral immune system tolerance [22,28]. In autoimmune diseases, there is dysregulation of the immune system where effector cells in the immunogenic arm become hyperreactive which is caused by a failure of immune system tolerance control. Tolerance is regulated at various checkpoints throughout the immune system [29]. Each checkpoint must create a balance to prevent autoimmunity but at the same time does not compromise immunity. Although the mechanism is not fully understood, autoimmunity is hypothesized to be caused by genetic susceptibility, damage to natural tolerance mechanisms, and environmental triggers such as infection [30,31].

### 2.1. Central Tolerance

**Figure 1:**
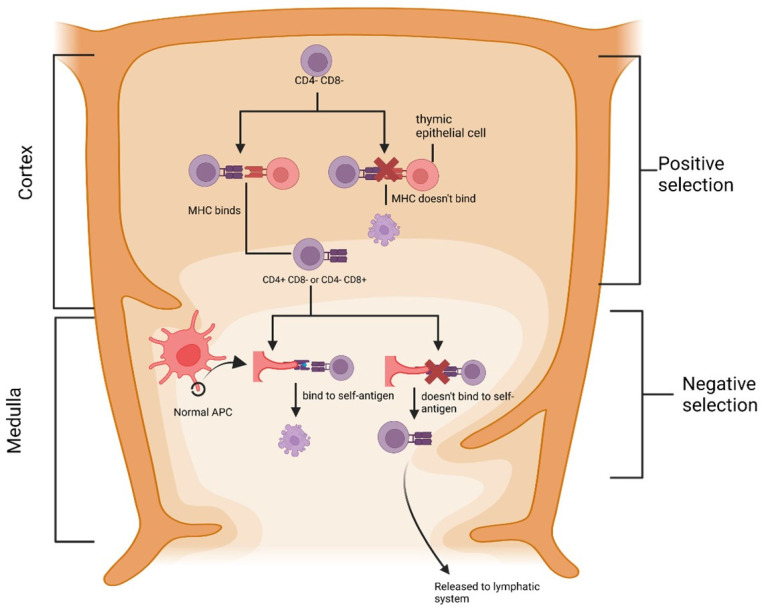
Establishment of central tolerance in the thymus. Double negative T-cells undergo positive selection by only cells bound to MHC of thymic epithelial cells that can survive. Subsequently, T-cells undergo negative selection, releasing only those that do not bind to self-peptide into circulation. MHC: major histocompatibility complex, CD4: cluster of fifferentiation-4, CD8: cluster of fifferentiation-8, and DC: dendritic cell.

The central tolerance mechanism is essential in forming tolerance and the first checkpoint of tolerance control by removing highly autoreactive lymphocytes. The formation of central tolerance occurs during the development of T cells in the thymus through a process of positive selection and negative selection [29]. Positive selection appears in the thymus cortex, where T-cell receptors that bind to MHCs in thymus epithelial cells can transport rapidly to the thymus medulla (Figure 1). On the other hand, the T cells in which the receptors cannot bind to the MHC do not survive.

In the thymus medulla, T cells that have undergone positive selection will undergo negative selection. T cells that have differentiated into CD4 or CD8 (single positive) rapidly move to the thymus medulla and then scan medullary antigen-presenting cells, dendritic cells (DC), and TEC (thymus epithelial cell) medullary cells (mTECs) for 4–5 days [28]. Thymocytes are removed if they have too high of an affinity for self-antigens (negative selection). Meanwhile, thymocytes with a low affinity for self-antigens will survive and exit the lymphoid circulation.

**Figure 2:**
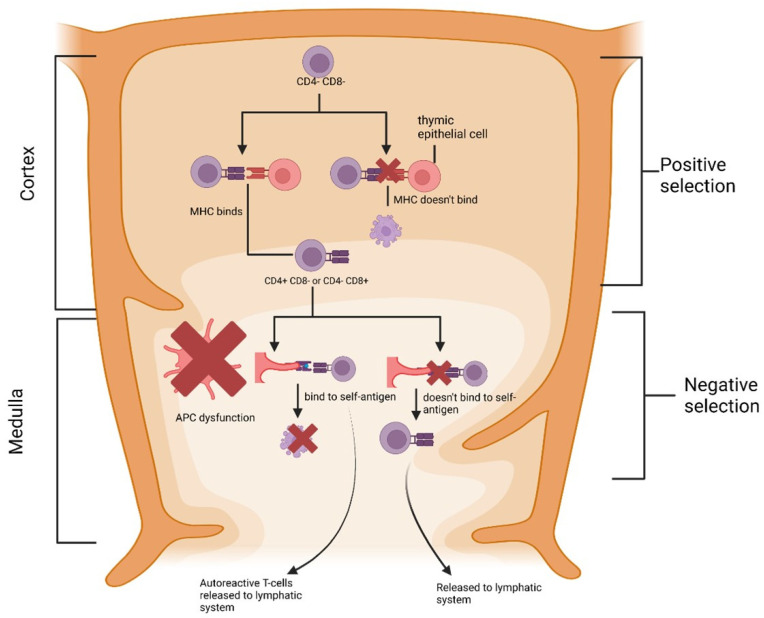
DC function abnormalities lead to failure of the formation of central tolerance in the thymus. Thymic DCs are involved in the presentation of self-peptide in T-cell development. Disturbance of self-peptide presentation allows self-reactive T-cells to be released into circulation. MHC: major histocompatibility complex, CD4: cluster of differentiation-4, CD8: cluster of differentiation-8, and DC: dendritic cells.

There are two possible fates of autoreactive T cells in the thymus: elimination through negative selection or differentiation into regulatory T cells [18]. The DC was shown to play a role in the differentiation of regulatory T cells in this thymus. Peripheral DCs can also migrate to the thymus, expose peripherally expressed self-antigens, and cause T cells to differentiate into regulatory T cells [32]. The DC also has surface molecules that are regulatory T-cell differentiation signals (CD70, CD80/86, ICOS-L, PD-L1, and PD-L2) [18]. Therefore, disruption of the DC results in abnormalities in central tolerance. This condition is the underlying cause of autoimmune diseases (Figure 2).

APCs in the thymus medulla (thymus medullary epithelial cells and DCs) express self-antigens specific to each organ tissue [28]. The autoimmune regulatory protein (AIRE) plays a vital role in activating this antigen expression. Losing just one AIRE-induced organ tissue-specific antigen in the thymus, can lead to autoimmunity in the target organ expressing that antigen [33]. The autosomal recessive AIRE gene mutation will cause autoimmune polyendocrine syndrome type-1 (APS-1) [33,34]. APS begins to appear in childhood and has various manifestations and can occur throughout an individual’s life with new manifestations that are difficult to predict. The most frequent manifestations are chronic mucocutaneous candidiasis, hypoparathyroidism, and primary adrenal insufficiency [35].

### 2.2. Peripheral Tolerance

**Figure 3:**
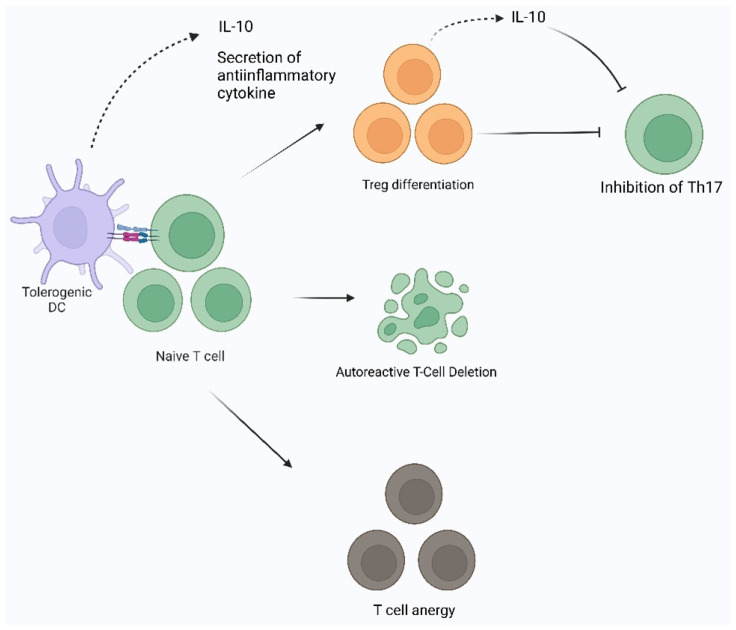
Establishment of peripheral tolerance by dendritic cells. TolDC can induce tolerance by direct secretion of anti-inflammatory cytokines, stimulate differentiation of Treg, induce apoptosis of self-reactive T-cells, and cause T-cell anergy. IL-10: interleukin-10, Th17:T-cell helper-17, and DC: dendritic cells.

DC also mediates the establishment of peripheral tolerances. In general, the main mechanisms of peripheral tolerance by DC are formed through the induction of clonal anergy, clonal deletion, metabolic modulation, and secretion of anti-inflammatory cytokines (Figure 3). T cells interacting strongly with tolDC can undergo anergy and lose their function. Some subsets of DC can induce anergy in T cells or the formation of regulatory T cells specific to the antigen being exposed [36]. tolDC also expresses cytotoxic T lymphocyte-associated antigen-4 (CTLA-4) and programmed cell death-1 (PD-1) which can inhibit T cell function. Peripheral autoantigens appear persistently, which will lead to clonal deletion [37]. The tolDC undergoes metabolic modulation by increasing mitochondrial oxidative activity and glycolysis capacity, producing ROS and superoxide [38]. An imbalance between ROS production and clearance leads to autoreactive T-cell damage and death [39]. In addition, tolDC expresses anti-inflammatory cytokines such as IL-10, TGF-β, and IL-35, so when tolDC is activated, it will prevent inflammation [40].

### 2.3. Role of Dendritic Cells in SLE

One autoimmune disease that arises due to peripheral tolerance abnormalities is SLE. Immunopathologically, SLE occurs due to a disturbance in peripheral tolerance where there is a disturbance in the elimination of apoptotic or necrotic body cells [12]. This causes the release of self-DNA/RNA, which will stimulate plasmacytoid DC (pDC), producing interferon type I (IFN-I) [41]. This increase in IFN-I production will induce an immunogenic immune response. DC differentiation occurs, increasing proinflammatory cytokines. The DC will present a self-antigen so that autoreactive T and B lymphocytes are formed [42].

Cellular death is an essential and innate occurrence observed in both regular bodily functions and abnormal states across all biological tissues. It is pivotal in maintaining immune tolerance and regulating the appropriate immune responses to external antigens. While apoptosis stands as the primary pathway for cellular death, cells can also undergo death via necrosis and necroptosis, which is a programmed cell death mechanism not reliant on caspase activation. Rapid removal of apoptotic cells from tissues is crucial to prevent inflammation and immune reactions. Inadequate clearance of apoptotic cells and subsequent buildup of cellular debris result in the release of autoantigens thus disrupting self-tolerance mechanisms [43]. Nuclear contents exposure coupled with persistence of danger signals that result from delayed apoptotic clearance and secondary necrosis result in the breakdown of tolerance to self-antinuclear antigens. Expression of ANA is not a protective mechanism to enhance phagocytosis, but rather pathological. The expression of ANA can be categorized as pathological insofar as antibodies specific to the disease are present in patients but not in otherwise healthy individuals; as such, the production of these antibodies signifies a pathological disturbance in the mechanisms that should prevent B cell and T cell reactivity to self-antigens [44]. Restoring tolerance towards the ANA antibody should reverse the process mediated by apoptotic clearance disturbance, and eventually should result disease remission.

In addition, SLE patients have an increased number and activity of Th 17 cells accompanied by a decrease in regulatory T cells, which causes autoimmunity. Furthermore, there is Th1 cell dysfunction and Th2 cell hyperfunction, which causes activation of various types of B cells, resulting in autoantibodies [45]. The formed complex of antigens and autoantibodies will cause damage to multiple tissues. This disruption of the innate and adaptive immune system occurs continuously. This causes tolDC to be unable to compensate for inflammatory activity caused either through anergy induction, clonal removal, metabolic modulation, or secretion of anti-inflammatory cytokines. Thus, increased tolDC capacity can restore the balance of immune tolerance regulation in SLE patients.

## 3. Recent Developments in Ex Vivo-Generated Tolerogenic Dendritic Cell Therapy for Autoimmune Diseases

In the last decade, much research has been carried out on tolDC as a potential target for immunotherapy in autoimmune patients. Similar to other autoimmune diseases, therapy in SLE aims to restore immune tolerance to self-antigens. Current clinical treatment of SLE uses anti-inflammatory and immunosuppressive drugs, which are nonspecific and have a broad systemic effect. Several new SLE therapy approaches are being developed to improve specificity and efficacy with fewer side effects. Therefore, tolDC is becoming a promising target and therapeutic tool for its ability to modulate immune responses to specific antigens. Cellular therapy with ex vivo induction of tolDC raises some interest in being developed for autoimmune therapies, especially SLE. Therefore, some preclinical studies focusing on tolDC transfer as an autoimmune disease therapy are summarized in Table 1.

TolDC’s autologous-based therapy is currently in clinical trials for treating several autoimmune diseases, including T1DM, rheumatoid arthritis, and multiple sclerosis [54,55,56,57]. However, little information is available regarding the use of tolDC for SLE therapy. There have been no clinical trials of SLE therapy using ex vivo-induced autologous DC. Some of the studies that have been conducted have only tested its function in vitro [46,47]. MoDC from SLE patients cultured with vitamin D and dexamethasone can produce IL-10-producing tolDC that induces a strong Treg response [37]. Another study showed that MoDC from SLE patients cultured with rosiglitazone and dexamethasone was not fully mature, characterized by low production of proinflammatory cytokines, and exhibited tolerogenic phenotypes. In addition, tolDC is resistant to autologous apoptotic cell-induced maturation and can decrease CD4 T cell responses [58].

**Figure 4:**
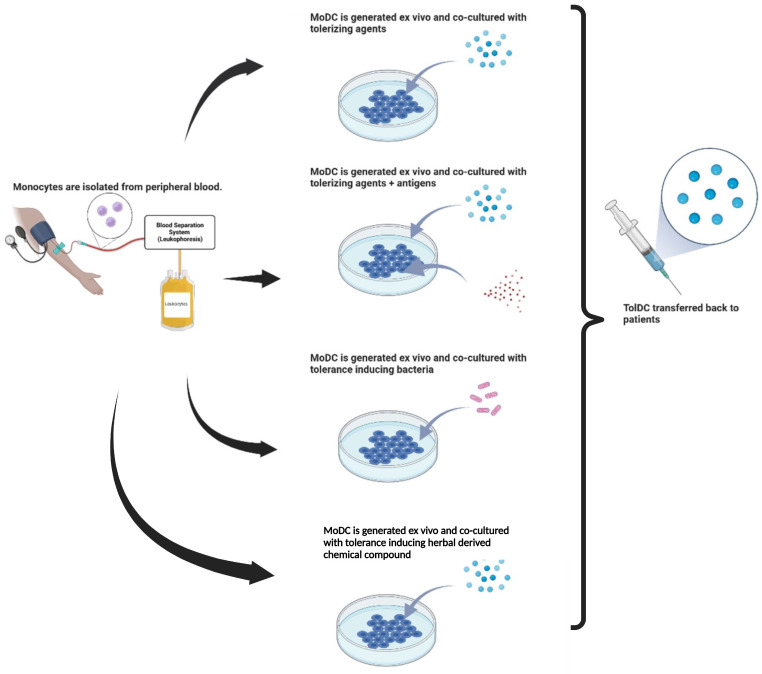
Production of tolDC for SLE. A. Ex vivo generation of tolDC can be done through various methods: co-culture with tolerizing agents, co-culture with tolerizing agents and specific antigens, co-culture with tolerance-inducing bacteria, and co-culture with tolerance inducing herbal-derived chemical compound.

Some probiotic bacteria show beneficial effects in lowering inflammation and autoimmune diseases. For example, moDCs of SLE patients cultured with probiotics (*Lactobacillus delbrueckii* and *Lactobacillus rhamnosus*) produced tolDC with low maturation rates. Furthermore, it was discovered that the procedure significantly increased the expression of IDO and IL-10 and decreased IL-12 in DC [59]. These results prove that tolDC can be made from peripheral blood monocytes of SLE patients cultured together with probiotic bacteria. Some of the currently developed tolDC manufacturing methods are illustrated in Figure 4.

Interestingly, Nikpoor et al. found that co-culture of MoDC derived from SLE patients with natural compounds derived from medicinal plants (curcumin and berberine) induced a tolerogenic phenotype in DC. These compounds suppress the maturation signal, decrease IL-12, and increase IL-10 production. These findings open the possibility of using such compounds in the ex vivo expansion of tolDC. The use of natural compounds might reduce the cost of tolDC production, so that other natural compounds should also be studied.

Several clinical trials of ex vivo-generated DC immunotherapy for autoimmune diseases have been conducted, summarized in Table 2. Administration of DC immunotherapy exposed intraarticularly with autologous synovial fluid in subjects with rheumatoid arthritis (RA) demonstrates good safety, tolerability, and efficacy. This is indicated by no flares and increased disease severity during the observation period [54]. Phase I clinical trials with T1DM subjects showed that DC immunotherapy was tolerable and had no serious adverse events. However, the study’s results did not show any clinical changes in the subjects treated [55]. Another study on T1DM subjects showed DC immunotherapy introduced with pancreatic islet cell antigens proved to be safe and well tolerated, induced an immune tolerance response for up to 3 years post-therapy, temporarily decreased CD4+ and CD8+ T cell responses to pancreatic islet cell autoantigens, and increased reg and memory CD4+ T cells after the first injection [57]. Phase IB clinical trials of DC immunotherapy in MS showed that patients given DC did not experience worsening during the follow-up phase. In addition, an increase in IL-10 was associated with an increase in regulator T cells [56]. Until now, there have been no clinical trial results of DC immunotherapy for SLE therapy. The results of the clinical trials mentioned above show that DC immunotherapy can be performed in autoimmune patients with reasonable safety and potential effectiveness. Therefore, DC immunotherapy should also be used for SLE therapy, but it still requires further research.

## 4. Therapeutic Potential, Problem, and Challenges of Dendritic Cell Immunotherapy for Autoimmune Diseases

Knowledge of immune mechanisms based on autoimmune diseases fueled the development of various approaches to induce tolDC. As a result, tolDC is expected to suppress unwanted immune responses in the long term and restore systemic immune tolerance. In addition, autologous DC transfers are proven to have high tolerability and do not cause unwanted side effects [54,55,56,57]. Thus, this method can be a long-term therapy for autoimmune diseases, including SLE.

Several methods are developed to induce the tolerogenic phenotype of DC ex vivo. TolDC can be obtained by culturing DC with an immunosuppressive agent, anti-inflammatory cytokine, or probiotic [37]. TolDC can also be obtained from genetic manipulation using viral vectors to express immunosuppressive phenotypes, such as the introduction of *CTLA-4* and *IDO* genes [60]. Although there are various potential ways to obtain tolDC, it is necessary to compare its effectiveness in inducing a tolerance response in autoimmune diseases, especially in SLE. In SLE, chronic inflammatory conditions occur, allowing changes in the tolDC phenotype to be autoreactive after being transferred to the patient’s body. For tolDC to effectively control autoimmunity in SLE, tolDC is needed to maintain its tolerogenic phenotype under inflammatory conditions.

DC can induce specific tolerance to antigens. Thus, introducing autoantigens by tolDC is expected to improve central and peripheral tolerance. However, it is still challenging to identify the exact specific antigen for systemic tolerance induction to occur. In autoimmune diseases that attack specific organs, part of the organ can be a source of autoantigens. This has been proven in clinical trials using synovial fluid as a source of autoantigens for DC therapy in rheumatoid arthritis [54]. However, in systemic autoimmune diseases involving multiple organs, such as SLE, using parts of a particular organ as a source of antigens may not represent the autoantigens that cause SLE.

It is known that SLE occurs due to impaired elimination of cells that undergo apoptosis or necrosis where self-DNA or RNA, histone, and nucleosomes trigger autoimmune reactions [20,61]. Preclinical trials using histone protein as a therapy showed promising results in reducing clinical symptoms, although no tolerance induction through Treg formation or anergy formation was proven [62]. Therefore, the design of specific antigens to induce tolerance in SLE is a challenge to be solved.

On the other hand, there is evidence that tolDCs exposed to specific antigens (loaded tolDC) have a lower capacity to induce tolerance compared to DCs not exposed to antigens (unloaded tolDC) [63]. The use of unloaded tolDCs may be more effective in systemic autoimmune diseases such as SLE because autoantigens are continuously present systemically in the body, so there is no need to be exposed to specific antigens ex vivo. Research on mice models of T1DM has also shown that unloaded tolDCs can trigger antigen-independent reg T cell expansion [53]. However, it should be noted that unloaded tolDC has an unstable phenotype, so there is a possibility that the phenotype changes to immunogenic due to exposure to antigens in the body [24]. If this happens, it will aggravate the autoimmunity reaction. Thus, it is necessary to study the direction of change in phenotype and functionality of unloaded tolDCs after it is transferred back to the body of patients with autoimmune diseases. In addition, it is also necessary to find methods to maintain the unloaded tolerogenic properties of tolDCs.

Regarding clinical applications, using immunosuppressant agents for SLE can cause different side effects [7,8]. However, some preclinical research in the SLE model shows the potential use of DC to initiate an immune tolerance response without causing significant side effects [46,47,49,51,52,53,64]. Therefore, the use of DC therapy is superior compared to standard treatment. However, the DC administration line still needs special attention. There is evidence that intraarticular administration of tolDC in rheumatoid arthritis can reduce symptoms [54], but this procedure tends to be invasive, making it challenging to give repeated doses.

Given the nature of the relapsing course of autoimmune diseases, it is possible that tolDC therapy needs to be given at repeated doses. Therefore, the DC administration route should be easily accessible and non-invasive but allow effective migration of DC to the lymphoid organs so that systemic immune tolerance can be induced.

Although no approach has yet been found to fully restore immune tolerance, autologous DC transfer could potentially replace or decrease dependence on immunosuppressant agents in people with SLE.

## 5. Conclusions

In summary, DC is vital in inducing central and peripheral immune tolerance. Abnormalities in DC function can lead to autoimmune diseases, including SLE, making DC one of the potential therapeutic targets. Preclinical studies revealed that tolDC can be generated ex vivo by culturing MoDC with differentiation media in combination with tolerizing agents (with or without antigens), tolerance-inducing bacteria, and herbal-derived chemical compound. Clinical studies revealed that tolDC is safe and well tolerated; however, clinical response is varied. Furthermore, identifying specific antigens, tolDC induction methods, and administrative pathways of DC immunotherapy for autoimmune diseases still needs further research.

## Figures and Tables

**Table 1:** Preclinical research of tolDC as an autoimmune therapy.

TolDC Induction Method	Study Model	Antigen Loading/Maturation Stimulus	Result	References
Ex vivo, MoDC differentiation media in combination with 1,25 dihydroxyvitamin D3+ Dexamethazone	In vitro model of MoDCs isolated from SLE patients	Lipopolysaccharide	Induces regulatory T cells and modulates cytokines	[46]
Ex vivo, MoDC differentiation media in combination with Rosiglitazone + Dexamethasone	In vitro model of MoDCs isolated from SLE patients	Lipopolysaccharide + Autologous Apoptotic Lymphocytes	Suppresses T cell priming and modulates cytokines	[47]
Ex vivo, either MoDC differentiation media Only (GM-CSF+IL-4) or combination with P Selectin or PD-1 or IL-10	In vitro model of MoDCs isolated from SLE patients	None	Diminished maturation signals expression, increased capability to induce Th17 cells, combination with IL-10 induces most potent regulatory T cells	[48]
Ex vivo, MoDC differentiation media in combination with *Lactobacillus delbruekii* + *Lactobacillus rhamnosus*	In vitro model of MoDCs isolated from SLE patients	None	Suppresses maturation signal expression, increases IDO and IL-10, and decreases IL-12	[49]
Ex vivo, MoDC differentiation media in combination with Curcumin or Berberin	In vitro model of MoDCs isolated from SLE patients	LPS	Suppresses maturation signal expression, decreases IL-12, increases IL-10	[50]
Ex vivo, MoDC differentiation media in combination with 1,25 dihydroxy vitamin D3	Mice model of multiple Sclerosis	None	Increase the proportion of regulatory T cells, CD4+IL-10+ T cells, and regulatory B cells in the spleen, reducing the infiltration of Th1 and Th17 cells into the spinal cord	[51]
Ex vivo transfection with plasmid lentivirus resulting in excessive expression of 25-hydroxyvitamin D 1α-hydroxylase	Mice model of multiple sclerosis	Myelin Antigens	Induces Th2, Treg 1, and T reg Foxp3+ in peripheral lymphoid tissue, suppressing specific symptoms of myelin	[52]
Ex vivo, MoDC differentiation medium Only (GM-CSF+IL-4)	Mice model of T1DM	Absence or antigen of pancreatic β cells	Inhibition of the progression of diabetes symptoms regardless of antigens, increasing proliferation of T regulator cells	[53]

**Table 2:** TolDC clinical research as an autoimmune disease therapy.

TolDC Induction Method	Disease	Antigen Loading/Maturation Stimulus	Administration	Clinical Trial Phase	Result	Challenge
Ex vivo MoDC Autolog	Rheumatoid Arthritis [54]	Autologous synovial fluid	Intraarticular	Ia	No immunomodulating effect or systemic effect. Improved symptoms were obtained in two out of three subjects.	Doses are given based on the protocol used for cancer because there have been no reports of tolDC administration for inflammatory arthritis. In addition, whether the intraarticular route can trigger systemic tolerance effects is unknown. Finally, the intraarticular route is invasive, making it undesirable for multiple dosages.
Ex vivo MoDC Autolog	T1DM [55]	None	Intradermal	I	Safe and well-tolerated	It cannot induce a systemic immunosuppressive response.
Ex vivo MoDC Autolog	Multiple sclerosis+Neuromyelitis Optica [56]	Some Myelin Proteins +AQP4	Intravenous	Ib	Safe and well tolerated. Increased Treg1 and anti-inflammatory cytokines (IL-10) were obtained.	The specific antigen target in multiple sclerosis is still unknown.
Ex vivo MoDC Autolog	T1DM [57]	Proinsulin peptide C19-A3	Intradermal	I	Safe and well tolerated. Induces an immune tolerance response for up to 3 years, a transient decrease in CD4+ and CD8+ T cell responses to pancreatic islet cell autoantigens, and increases T reg cells and memory CD4+ T cells after the first injection.	

## Data Availability

All data is available upon request.

## References

[1] Katsarou A., Gudbjörnsdottir S., Rawshani A., Dabelea D., Bonifacio E., Anderson B.J., Jacobsen L.M., Schatz D.A., Lernmark Å. (2017). Type 1 diabetes mellitus. Nat. Rev. Dis. Primers.

[2] Filippi M., Bar-Or A., Piehl F., Preziosa P., Solari A., Vukusic S., Rocca M.A. (2018). Multiple sclerosis. Nat. Rev. Dis. Primers.

[3] Smolen J.S., Aletaha D., Barton A., Burmester G.R., Emery P., Firestein G.S., Kavanaugh A., McInnes I.B., Solomon D.H., Strand V. (2018). Rheumatoid arthritis. Nat. Rev. Dis. Primers.

[4] Ameer M.A., Chaudhry H., Mushtaq J., Khan O.S., Babar M., Hashim T., Zeb S., Tariq M.A., Patlolla S.R., Ali J. (2022). An Overview of Systemic Lupus Erythematosus (SLE) Pathogenesis, Classification, and Management. Cureus.

[5] Pisetsky D.S. (2023). Pathogenesis of autoimmune disease. Nat. Rev. Nephrol..

[6] Morand E.F., Fernandez-Ruiz R., Blazer A., Niewold T.B. (2023). Advances in the management of systemic lupus erythematosus. BMJ.

[7] Deng J., Chalhoub N.E., Sherwin C.M., Li C., Brunner H.I. (2019). Glucocorticoids pharmacology and their application in the treatment of childhood-onset systemic lupus erythematosus. Semin. Arthritis Rheum..

[8] Quan X.-Y., Chen H.-T., Liang S.-Q., Yang C., Yao C.-W., Xu Y.-Z., Liu H.-F., An N. (2022). Revisited Cyclophosphamide in the Treatment of Lupus Nephritis. BioMed. Res. Int..

[9] Mosanya C.H., Isaacs J.D. (2018). Tolerising cellular therapies: What is their promise for autoimmune disease?. Ann. Rheum. Dis..

[10] Hilkens C.M.U., Isaacs J.D. (2013). Tolerogenic dendritic cell therapy for rheumatoid arthritis: Where are we now?. Clin. Exp. Immunol..

[11] Passeri L., Marta F., Bassi V., Gregori S. (2021). Tolerogenic Dendritic Cell-Based Approaches in Autoimmunity. Int. J. Mol. Sci..

[12] Arneth B. (2019). Systemic Lupus Erythematosus and DNA Degradation and Elimination Defects. Front. Immunol..

[13] Herrmann M., Podolska M., Biermann M., Maueröder C., Hahn J. (2015). Inflammatory etiopathogenesis of systemic lupus erythematosus: An update. J. Inflamm. Res..

[14] Ahlers M.J., Lowery B.D., Farber-Eger E., Wang T.J., Bradham W., Ormseth M.J., Chung C.P., Stein C.M., Gupta D.K. (2020). Heart Failure Risk Associated With Rheumatoid Arthritis–Related Chronic Inflammation. J. Am. Hear. Assoc..

[15] AlFaris H., Alkathiri S.A., Babelli D., Alokaily F. (2022). Double malignancy and a mycobacterial infection in a rheumatoid arthritis patient. SciVee.

[16] Johnson D.K., Reynolds K.M., Poole B.D., Montierth M.D., Todd V.M., Barnado A., Davis M.F. (2021). Contribution of viral infection to risk for cancer in systemic lupus erythematosus and multiple sclerosis. PLoS ONE.

[17] Song M., Latorre G., Ivanovic-Zuvic D., Camargo M.C., Rabkin C.S. (2019). Autoimmune diseases and gastric cancer risk: A systematic review and meta-analysis. Cancer Res. Treat..

[18] Waisman A., Lukas D., Clausen B.E., Yogev N. (2016). Dendritic cells as gatekeepers of tolerance. Semin. Immunopathol..

[19] Balan S., Saxena M., Bhardwaj N. (2019). Dendritic Cell Subsets and Locations.

[20] Saadeh D., Kurban M., Abbas O. (2016). Update on the role of plasmacytoid dendritic cells in inflammatory/autoimmune skin diseases. Exp. Dermatol..

[21] Takagi H., Arimura K., Uto T., Fukaya T., Nakamura T., Choijookhuu N., Hishikawa Y., Sato K. (2016). Plasmacytoid dendritic cells orchestrate TLR7-mediated innate and adaptive immunity for the initiation of autoimmune inflammation. Sci. Rep..

[22] Kisielow P. (2019). How does the immune system learn to distinguish between good and evil? The first definitive studies of T cell central tolerance and positive selection. Immunogenetics.

[23] Macri C., Pang E.S., Patton T., O’Keeffe M. (2018). Dendritic cell subsets. Semin. Cell Dev. Biol..

[24] Kim M.K., Kim J. (2019). Properties of immature and mature dendritic cells: Phenotype, morphology, phagocytosis, and migration. RSC Adv..

[25] Ugur M., Mueller S.N. (2019). T cell and dendritic cell interactions in lymphoid organs: More than just being in the right place at the right time. Immunol. Rev..

[26] Hilligan K.L., Ronchese F. (2020). Antigen presentation by dendritic cells and their instruction of CD4+ T helper cell responses. Cell. Mol. Immunol..

[27] Roche P.A., Cresswell P. (2016). Antigen processing and presentation mechanisms in myeloid cells. Microbiol. Spectr..

[28] Klein L., Hinterberger M., Wirnsberger G., Kyewski B. (2009). Antigen presentation in the thymus for positive selection and central tolerance induction. Nat. Rev. Immunol..

[29] Murphy K., Weaver C. (2017). Janeway’s Immunobiology.

[30] Czaja A.J., Santrach P.J., Moore S.B. (2001). Shared Genetic Risk Factors in Autoimmune Liver Disease. Dig. Dis. Sci..

[31] Nielsen P.R., Kragstrup T.W., Deleuran B.W., Benros M.E. (2016). Infections as risk factor for autoimmune diseases—A nationwide study. J. Autoimmun..

[32] Proietto A.I., van Dommelen S., Zhou P., Rizzitelli A., D’Amico A., Steptoe R.J., Naik S.H., Lahoud M.H., Liu Y., Zheng P. (2008). Dendritic cells in the thymus contribute to T-regulatory cell induction. Proc. Natl. Acad. Sci. USA.

[33] Metzger T.C., Anderson M.S. (2011). Control of central and peripheral tolerance by Aire. Immunol. Rev..

[34] Bruserud Ø., Oftedal B.E., Wolff A.B., Husebye E.S. (2016). AIRE-mutations and autoimmune disease. Curr. Opin. Immunol..

[35] Borchers J., Pukkala E., Mäkitie O., Laakso S. (2020). Patients with APECED Have Increased Early Mortality Due to Endocrine Causes, Malignancies and infections. J. Clin. Endocrinol. Metab..

[36] Ning B., Wei J., Zhang A., Gong W., Fu J., Jia T., Yang S.-Y. (2015). Antigen-specific tolerogenic dendritic cells ameliorate the severity of murine collagen-induced arthritis. PLoS ONE.

[37] Funes S.C., de Lara A.M., Altamirano-Lagos M.J., Mackern-Oberti J.P., Escobar-Vera J., Kalergis A.M. (2019). Immune checkpoints and the regulation of tolerogenicity in dendritic cells: Implications for autoimmunity and immunotherapy. Autoimmun. Rev..

[38] Malinarich F., Duan K., Hamid R.A., Bijin A., Lin W.X., Poidinger M., Fairhurst A.-M., Connolly J.E. (2015). High Mitochondrial Respiration and Glycolytic Capacity Represent a Metabolic Phenotype of Human Tolerogenic Dendritic Cells. J. Immunol..

[39] Peng H.-Y., Lucavs J., Ballard D., Das J.K., Kumar A., Wang L., Ren Y., Xiong X., Song J. (2021). Metabolic Reprogramming and Reactive Oxygen Species in T Cell Immunity. Front. Immunol..

[40] Dixon K.O., van der Kooij S.W., Vignali D.A.A., van Kooten C. (2015). Human tolerogenic dendritic cells produce IL-35 in the absence of other IL-12 family members. Eur. J. Immunol..

[41] Pan L., Lu M.-P., Wang J.-H., Xu M., Yang S.-R. (2019). Immunological pathogenesis and treatment of systemic lupus erythematosus. World J. Pediatr..

[42] Kaewraemruaen C., Ritprajak P., Hirankarn N. (2020). Dendritic cells as key players in systemic lupus erythematosus. Asian Pac. J. Allergy Immunol..

[43] Shao W.-H., Cohen P.L. (2010). Disturbances of apoptotic cell clearance in systemic lupus erythematosus. Arthritis Res. Ther..

[44] Pisetsky D.S., Lipsky P.E. (2020). New insights into the role of antinuclear antibodies in systemic lupus erythematosus. Nat. Rev. Rheumatol..

[45] Chen P.-M., Tsokos G.C. (2021). T Cell Abnormalities in the Pathogenesis of Systemic Lupus Erythematosus: An Update. Curr. Rheumatol. Rep..

[46] Wu H.J., Lo Y., Luk D., Lau C.S., Lu L., Mok M.Y. (2015). Alternatively activated dendritic cells derived from systemic lupus erythematosus patients have tolerogenic phenotype and function. Clin. Immunol..

[47] Obreque J., Vega F., Torres A., Cuitino L., Mackern-Oberti J.P., Viviani P., Kalergis A., Llanos C. (2017). Autologous tolerogenic dendritic cells derived from monocytes of systemic lupus erythematosus patients and healthy donors show a stable and immunosuppressive phenotype. Immunology.

[48] Estrada-Capetillo L., Hernández-Castro B., Monsiváis-Urenda A., Alvarez-Quiroga C., Layseca-Espinosa E., Abud-Mendoza C., Baranda L., Urzainqui A., Sánchez-Madrid F., González-Amaro R. (2013). Induction of Th17 Lymphocytes and Treg Cells by Monocyte-Derived Dendritic Cells in Patients with Rheumatoid Arthritis and Systemic Lupus Erythematosus. J. Immunol. Res..

[49] Esmaeili S., Mahmoudi M., Rezaieyazdi Z., Sahebari M., Tabasi N., Sahebkar A., Rastin M. (2018). Generation of tolerogenic dendritic cells using *Lactobacillus rhamnosus* and *Lactobacillus delbrueckii* as tolerogenic probiotics. J. Cell. Biochem..

[50] Nikpoor A.R., Mahmoudi M., Shapouri-Moghaddam A., Rezaieyazdi Z., Mollazadeh S., Tabasi N., Mansouri A., Moghadam R.M., Momtazi A.A., Najmaldin S.K. (2024). Curcumin and Berberine Arrest Maturation and Activation of Dendritic Cells Derived from Lupus Erythematosus Patients. Curr. Mol. Pharmacol..

[51] Xie Z., Chen J., Zheng C., Wu J., Cheng Y., Zhu S., Lin C., Cao Q., Zhu J., Jin T. (2017). 1,25-dihydroxyvitamin D_3_-induced dendritic cells suppress experimental autoimmune encephalomyelitis by increasing proportions of the regulatory lymphocytes and reducing T helper type 1 and type 17 cells. Immunology.

[52] Li C.H., Zhang J., Baylink D.J., Wang X., Goparaju N.B., Xu Y., Wasnik S., Cheng Y., Berumen E.C., Qin X. (2017). Dendritic cells, engineered to overexpress 25-hydroxyvitamin D 1α-hydroxylase and pulsed with a myelin antigen, provide myelin-specific suppression of ongoing experimental allergic encephalomyelitis. FASEB J..

[53] Lo J., Xia C.-Q., Peng R., Clare-Salzler M.J. (2018). Immature Dendritic Cell Therapy Confers Durable Immune Modulation in an Antigen-Dependent and Antigen-Independent Manner in Nonobese Diabetic Mice. J. Immunol. Res..

[54] Bell G.M., Anderson A.E., Diboll J., Reece R., Eltherington O., Harry R.A., Fouweather T., MacDonald C., Chadwick T., McColl E. (2016). Autologous tolerogenic dendritic cells for rheumatoid and inflammatory arthritis. Ann. Rheum. Dis..

[55] Giannoukakis N., Phillips B., Finegold D., Harnaha J., Trucco M. (2011). Phase I (Safety) Study of Autologous Tolerogenic Dendritic Cells in Type 1 Diabetic Patients. Diabetes Care.

[56] Zubizarreta I., Flórez-Grau G., Vila G., Cabezón R., España C., Andorra M., Saiz A., Llufriu S., Sepulveda M., Sola-Valls N. (2019). Immune tolerance in multiple sclerosis and neuromyelitis optica with peptide-loaded tolerogenic dendritic cells in a phase 1b trial. Proc. Natl. Acad. Sci. USA.

[57] Nikolic T., Suwandi J.S., Wesselius J., Laban S., Joosten A.M., Sonneveld P., Mul D., Aanstoot H.-J., Kaddis J.S., Zwaginga J.J. (2022). Tolerogenic dendritic cells pulsed with islet antigen induce long-term reduction in T-cell autoreactivity in type 1 diabetes patients. Front. Immunol..

[58] AHorwitz D. (2008). Regulatory T cells in systemic lupus erythematosus: Past, present and future. Arthritis Res. Ther..

[59] Mitchell D., Chintala S., Dey M. (2018). Plasmacytoid dendritic cell in immunity and cancer. J. Neuroimmunol..

[60] Tiberio L., Del Prete A., Schioppa T., Sozio F., Bosisio D., Sozzani S. (2018). Chemokine and chemotactic signals in dendritic cell migration review-article. Cell Mol. Immunol..

[61] Lorenz G., Anders H.J. (2015). Neutrophils, Dendritic Cells, Toll-Like Receptors, and Interferon-α in Lupus Nephritis. Semin. Nephrol..

[62] Funes S.C., Ríos M., Gómez-Santander F., Fernández-Fierro A., Altamirano-Lagos M.J., Rivera-Perez D., Pulgar-Sepúlveda R., Jara E.L., Rebolledo-Zelada D., Villarroel A. (2019). Tolerogenic dendritic cell transfer ameliorates systemic lupus erythematosus in mice. Immunology.

[63] Funda D.P., Goliáš J., Hudcovic T., Kozáková H., Špíšek R., Palová-Jelínková L. (2018). Antigen Loading (e.g., Glutamic Acid Decarboxylase 65) of Tolerogenic DCs (tolDCs) Reduces Their Capacity to Prevent Diabetes in the Non-Obese Diabetes (NOD)-Severe Combined Immunodeficiency Model of Adoptive Cotransfer of Diabetes As Well As in NOD Mice. Front. Immunol..

[64] Paiatto L.N., Silva F.G.D., Yamada T., Tamashiro W.M.S.C., Simioni P.U. (2018). Adoptive transfer of dendritic cells expressing CD11c reduces the immunological response associated with experimental colitis in BALB/c mice. PLoS ONE.

